# Feasibility of a pilot randomized controlled trial on cranial remolding orthosis for severe deformational plagiocephaly

**DOI:** 10.33137/cpoj.v9i1.46298

**Published:** 2026-01-28

**Authors:** P Amir-Yazdani, R Alhaeik, M Gimeno, K Fontaine, A Bursuc, A.G Weil, C.K Costa

**Affiliations:** 1 Department of Pediatrics, Division of Physical Medicine and Rehabilitation, Faculty of Medicine, Centre hospitalier universitaire Sainte-Justine, Montreal, Canada.; 2 Faculty of Medicine, University of Montreal, Montreal, Canada.; 3 Service des aides techniques, Centre hospitalier universitaire Sainte-Justine, Montreal, Canada.; 4 Division of Clinical and Translational Research, Faculty of Medicine and Health Sciences, McGill University, Montreal, Canada.; 5 Division of Neurosurgery, Faculty of Medicine, Centre hospitalier universitaire Sainte-Justine, Montreal, Canada.; 6 Faculty of Medicine, McGill University, Montreal, Canada.; 7 Shriners Hospital for Children, Montreal, Canada.

**Keywords:** Deformational Plagiocephaly, Cranial Remolding Orthosis, Helmet Therapy, Head Protective Devices, Infant Head Shape, Randomized Controlled Trial, Feasibility Study, Cranial Asymmetry, Parental Satisfaction, Orthotic Devices, Plagiocephaly, Pediatric Rehabilitation

## Abstract

**BACKGROUND:**

Prevalence of deformational plagiocephaly (DP) has increased since the “Back to sleep” campaign to counter sudden infant death syndrome. Cranial remolding orthosis (CRO) is commonly used to manage severe DP in infants, yet evidence from randomized controlled trials (RCT) remains limited.

**OBJECTIVES:**

This pilot RCT aimed to assess the feasibility of a future large-scale RCT on cranial remolding orthosis for severe DP in infants aged 4 to 7 months. Specific objectives included evaluating recruitment, retention, coordination, and parental acceptability. A secondary objective was exploring trends in cranial shape outcomes between groups over 6- and 12-week periods.

**METHODOLOGY:**

Seventy-eight infants were screened at a single pediatric tertiary-care center between December 2023 and June 2024, and 31 families were approached for consent after initial clinical evaluation. Following consent, head shape severity was assessed using three-dimensional surface imaging, and seven infants were excluded prior to randomization due to insufficient severity. Twenty-four infants were randomized to receive immediate (n = 12) or delayed (six-week delay) (n = 12) CRO. One participant randomized to the delayed arm received the orthosis earlier than planned due to a protocol deviation. Feasibility metrics included recruitment timelines, protocol adherence, appointment coordination, and parental acceptability. Exploratory efficacy analyses evaluated cranial vault asymmetry (CVA), cranial vault asymmetry index (CVAI), oblique diameter diagonal difference (ODDI), cranioproportional index (CI), Argenta severity scores, and parent-reported head shape perception and satisfaction.

**FINDINGS:**

Of the 24 randomized infants, 20 were male and four were female. Mean age at enrollment was 5.3 ± 0.9 months in the intervention arm and 5.1 ± 0.9 months in the control arm. Recruitment and retention targets were met within seven months despite scheduling challenges. Parental satisfaction was high (mean score of 4.5 ± 0.1 on a 5-point scale), and all families reported willingness to repeat treatment despite common but minor side effects, including sweating, orthosis odor, and mild skin irritation. Significant improvements over time were observed in CVA, ODDI, and CI (all p < 0.001), with no significant between-group differences observed at 6 or 12 weeks.

**CONCLUSION:**

This pilot trial confirmed the feasibility and acceptability of conducting a larger RCT on CRO for DP, demonstrating successful recruitment, retention, and protocol implementation. Cranial symmetry improved over time in both groups, with no statistically significant differences observed between immediate and delayed treatment arms over the 12-week period. Future larger studies are needed to assess clinical effectiveness and should consider broader inclusion criteria, refined measurement techniques, and dedicated coordination to address scheduling challenges and ensure rigorous implementation and generalizability.

## INTRODUCTION

Deformational plagiocephaly (DP) is an asymmetric cranial shape in the absence of craniosynostosis characterized by unilateral parieto-occipital flattening.^1^ The 1992 “Back to Sleep” campaign by the American Academy of Pediatrics advocated for supine sleeping and firmer infant mattresses to reduce sudden infant death syndrome (SIDS).^2,3^ These recommendations reduced SIDS but contributed to increased positional cranial deformities.^4^

Beyond cosmetic implications, DP has been associated with lower quality of life and dental malocclusions.^5–7^ It may also co-occur with delayed gross motor development, either in isolation or alongside global or cognitive developmental delay.^8,9^

Management may include observation, as cranial remodeling occurs with growth and decreased time spent supine. However, in severe cases, spontaneous improvement may be limited.^10^ Physiotherapy can correct underlying causes such as torticollis and improve motor milestone acquisition.^11,12^ Parental positioning strategies can reduce asymmetry but are generally less effective than cranial remolding orthosis (CRO).^13,14^ A systematic review highlighted substantial non-randomized evidence supporting CRO for persistent moderate to severe plagiocephaly after conservative treatment, and for older infants with moderate to severe deformity.^15^

The HElmet therapy Assessment in infants with Deformed Skulls (HEADS) trial, an RCT of 84 infants aged 5 to 6 months, challenged the perceived effectiveness of CRO by reporting no significant advantage over natural progression through 24 months.^16^ However, its generalizability was limited by exclusion of premature infants, muscular torticollis, and very severe cranial deformities. It also reported unusually high rate of helmet fit issues (73%) compared with rates typically reported in the literature.^7^ This RCT influenced guidelines such as Choosing Wisely Canada and contributed to reduced public insurance coverage for CRO in some regions.^17^ Subsequent non-randomized studies have continued to support CRO, demonstrating sustained improvements in cranial asymmetry until age four.^7,18^ More recent reviews have highlighted persistent variability in study design, outcome measures, and patient selection, underscoring the need for well-designed prospective trials to clarify effectiveness and optimal treatment timing.^19^

At our institution, the CHU Sainte-Justine, physiatrists routinely prescribe CRO and observe positive clinical outcomes with minimal side effects. Eligible infants are offered a custom CRO fabricated in the hospital-owned orthotics laboratory, fully covered by public insurance, and are monitored by experienced orthotists.

This pilot study aimed to assess the feasibility of a future large-scale RCT on CRO for severe DP in infants aged 4 to 7 months. In this study, severe DP was defined clinically as an Argenta classification of III-V and confirmed using three-dimensional (3D) surface imaging, with thresholds of cranial vault asymmetry (CVA) ≥ 12 mm, cranial vault asymmetry index (CVAI) ≥ 12%, and/or oblique diameter difference index (ODDI) ≥ 112% (see Outcome measures). Specific objectives included evaluating recruitment, retention, coordination, and parental acceptability of the intervention. A secondary objective was to explore trends in cranial shape outcomes between intervention groups over time. We hypothesized that the study protocol would be feasible and acceptable by staff and parents, since it required little modification from the usual treatment course. We also hypothesized infants who started using the CRO earlier would present an enhanced rate of improvement in head shape.

## METHODOLOGY

This prospective, single-center, pilot RCT was conducted at the CHU Sainte-Justine, a pediatric university hospital in Montreal, Canada.

Recruitment occurred from December 2023 to June 2024, with final follow-up completed in September 2024. Data collection was completed in December 2024.

### Participants and Recruitment

All infants evaluated for DP in the physiatry outpatient clinics at the CHU Sainte-Justine during the recruitment period were considered for participation. At the initial visit, the senior investigator (Costa C.K) screened infants for age (4 to 7 months) and clinical severity (Argenta types III-V).

A research collaborator not involved in clinical care provided study information and obtained written informed consent. Following consent, cranial measurements were obtained by orthotists using 3D surface imaging and were analyzed by the primary investigator (Amir-Yazdani P). Final eligibility was confirmed using predefined cranial asymmetry thresholds (see Outcome measures). Infants not meeting these criteria were excluded prior to randomization. Recruitment, consent, measurements, and randomization occurred on the same day.

### Inclusion Criteria

- Age between 4 and 7 months (minus one day) at the time of the first clinical evaluation (corrected age for preterm infants).
- Evaluated by a physiatrist at the CHU Sainte-Justine plagiocephaly clinic.
- Diagnosis of severe DP, confirmed by 3D surface imaging (see Outcome measures).
- Parent or legal guardian could provide informed consent and communicate reliably.

### Exclusion Criteria

- Craniosynostosis.
- Isolated brachycephaly.
- Other craniofacial deformities or syndromes.

### Randomization and Blinding

Participants were randomized 1:1 using block randomization with variable block sizes. The sequence was generated in Microsoft Excel 2022 by a research assistant (Alhaeik R) uninvolved in clinical care. Allocation concealment was ensured using sequentially numbered, opaque, sealed envelopes opened by the primary investigator (Amir-Yazdani P) after eligibility confirmation and consent.

### Intervention

**Figure 1:**
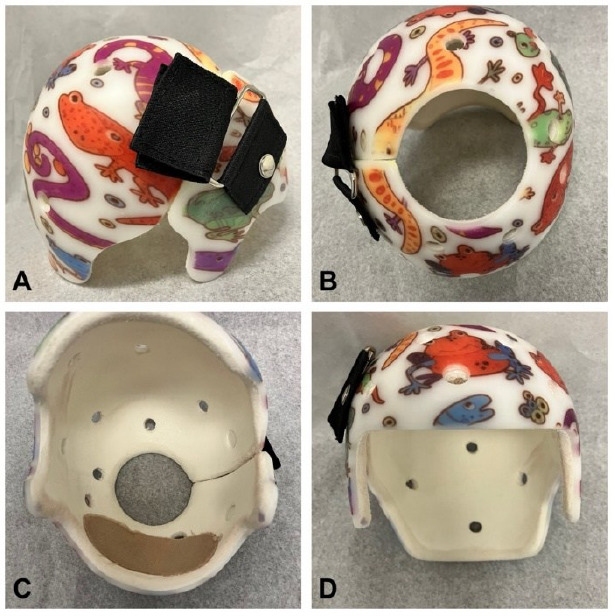
Example of a cranial remolding orthosis. (A) Lateral view; (B) superior view; (C) inferior view; (D) frontal view.

CRO was administered to treat DP (**Figure 1**). Orthoses were fabricated by experienced orthotists using 3D surface imaging (Rodin4D software) of cranial morphology. The image is manually adjusted to create a computer numerical control milled foam positive head model, upon which a thermoplastic shell is manually formed. The CRO provides a relief of maximum one centimeter in the plagiocephalic area, and Aliplast lines the thermoplastic shell for total contact of the rest of the infant’s cranium. Our standard of care involves follow-ups every six weeks, or more frequently as needed, to adjust for cranial growth. This technique has been used at our institution for over 20 years, evolving from earlier plaster-based methods.

Parents were instructed to aim for 23 hours of daily wear. Participants were included in the per-protocol (PP) analysis if reported wear time was ≥ 20 hours daily, in line with evidence that efficacy is proportional to wear time up to this threshold.^20^

Participants were randomized into two groups. The intervention arm received their orthosis one week after the initial physiatry visit. The control arm followed the standard institutional timeline and had their measurement for the cranial orthosis 6 weeks after initial physiatry visit. The delay in treatment initiation between groups aimed to allow comparison of cranial asymmetry evolution. Given the perceived effectiveness of cranial orthoses at our institution, withholding or significantly delaying treatment was deemed unethical.

### Outcome Measures

Feasibility outcomes were assessed using structured logs documenting barriers and facilitators reported by investigators, physicians, orthotists, administrative staff, and parents. Parental feedback was also obtained by questionnaire (Appendix A). Feasibility domains included recruitment and adherence to inclusion criteria, acceptability of randomization and assessment procedures, retention rates, coordination between physiatry clinics and orthotics clinics, and parental acceptability of CRO.

Various outcome measures were tested to detect changes in cranial asymmetry between intervention groups over time. These were derived from 3D surface imaging (Rodin4D software), and included CVA, CVAI, ODDI, and cranioproportional index (CI). Severity of deformity was clinically determined using the Argenta classification, with scores of III to V deemed as severe.^21^ Descriptions of outcome measures, severity thresholds, and references are provided in Appendix B.^16,25,26,29–34^ Cranial measurements were collected at predefined moments (**Table 1**).

**Table 1:** Timeline of Data Collection.

Timepoints	Intervention arm – Immediate orthosis	Control arm – Delayed orthosis
**Baseline**	First consult with physiatrist and assessment of Argenta scale. First visit with orthotist for 3D surface imaging.	First consult with physiatrist and assessment of Argenta scale. First visit with orthotist for 3D surface imaging.
**After 1 week**	Delivery of cranial orthosis.	–
**After 6 weeks**	Second visit with orthotist for 3D surface imaging (measurements post-5 weeks of treatment) with adjustments if needed.	Second visit with orthotist for 3D surface imaging (measurements post-6 weeks of observation and for fabrication of the cranial orthosis).
**After 7 weeks**	–	Delivery of cranial orthosis.
**After 12 weeks**	Third visit with orthotist for 3D surface imaging (measurements post-11 weeks of treatment). Second visit with the physiatrist for medical follow-up and assessment of Argenta scale.	Third visit with orthotist for 3D surface imaging (measurements post-5 weeks of treatment). Second visit with the physiatrist for medical follow-up and assessment of Argenta scale.

### Statistical Methods

Feasibility outcomes were assessed qualitatively and quantitatively. A root cause analysis identified barriers and facilitators, supported by quantitative triangulation including recruitment rates, appointment adherence, and missing data patterns.

Exploratory analyses of treatment effectiveness were conducted to evaluate trends and assess the suitability of planned statistical methods. Cranial shape measures (CVA, CVAI, ODDI, CI) were analyzed using repeated measures ANOVA with mixed models, incorporating time (within-subject) and group (between-subject) factors. Changes in Argenta scores were analyzed using Mann-Whitney U tests and chi-square (χ²) tests.

Parent-reported outcomes included head-shape perception and treatment satisfaction. Changes in head-shape perception scores from baseline to 12 weeks were assessed within each group using Wilcoxon signed-rank tests, and the magnitude of change was compared between groups using a Wilcoxon rank-sum test (Mann–Whitney U). Satisfaction with CRO at 12 weeks was compared between groups using a Wilcoxon rank-sum test.

All analyses were performed using R version 4.5.2 (R Foundation for Statistical Computing, Vienna, Austria). A two-tailed α level of 0.05 was used to define statistical significance.

Due to the nature of the intervention, blinding was not feasible for participants, parents or clinicians; however, outcome assessors remained blinded (single-blinded).

### Publication Ethics

This study was approved by the Research Ethics Committee of the CHU Sainte-Justine (approval number: 2024-5800). Written informed consent was obtained from all parents or legal guardians. The trial was registered at ClinicalTrials.gov (NCT06173102).

## RESULTS

The study targeted 24 participants, consistent with guidance suggesting that 12 subjects per group is sufficient for pilot studies intended to inform future trials.^22^ Registered trials include samples as small as 8 participants per arm.^23^ This sample size was chosen to assess feasibility rather than clinical efficacy.

Seventy-eight infants were screened during the recruitment period. Of these, 47 were not approached for consent because they did not meet prespecified eligibility criteria based on age or clinical severity at initial assessment. Thirty-one families were approached for consent, and none declined participation. Following consent, seven infants were excluded due to insufficient DP severity based on 3D surface imaging measurements.

Twenty-four participants were randomized equally to the intervention (n = 12) and control (n = 12) arms and included in the intention-to-treat (ITT) analysis. Missing data were handled using mixed-effects modeling, allowing inclusion of all available longitudinal data.

Two participants from the intervention arm did not meet the predefined wear threshold (≥ 20 hours/day) and were omitted from the PP analysis but retained in the ITT analysis. One protocol deviation occurred when a participant randomized to the control arm received the orthosis earlier than planned due to a scheduling error during appointment coordination, resulting in treatment initiation aligned with the intervention arm timeline. This participant was analyzed according to randomized allocation in the ITT analysis and according to treatment received in the PP analysis.

**Figure 2:**
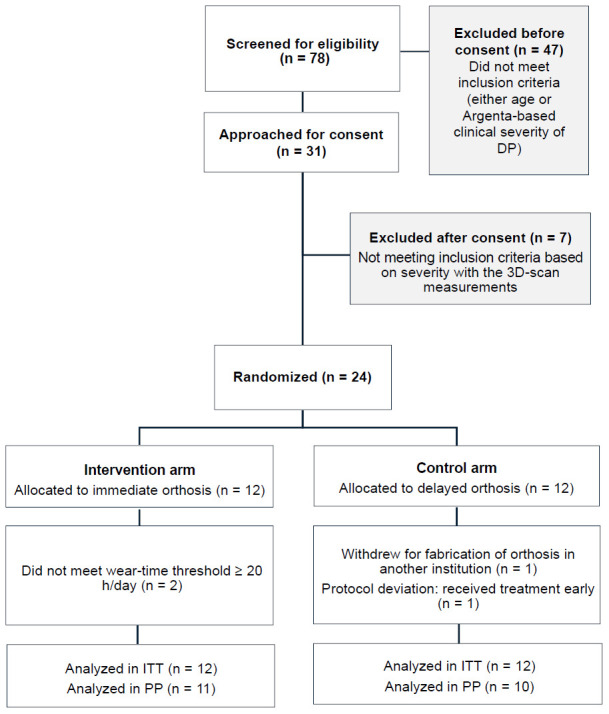
CONSORT flow diagram of participant recruitment and retention.

**Note:** Participants who did not meet the predefined wear-time threshold were included in the ITT analysis but excluded from the PP analysis. One participant withdrew before receiving allocated intervention and was included in the ITT analysis but excluded from the PP analysis. One participant randomized to the control arm received the orthosis earlier than planned due to a protocol deviation and was analyzed according to randomized allocation in the ITT analysis and according to treatment received in the PP analysis.

No participants were lost to follow-up; one control group participant withdrew prior to receiving the allocated intervention after obtaining orthosis treatment in another institution (**Figure 2**). Baseline characteristics are summarized in **Table 2**.

**Table 2:** Baseline clinical and demographic characteristics.^1^

		Intervention arm (n=12)	Control arm (n=12)
**Sex**	Male	12 (100%)	8 (67%)
	Female	-	4 (33%)
**Age at enrolment (months)**		5.3 ± 0.9	5.1 ± 0.9
**Birth rank^2^**	1	9 (75%)	6 (54%)
	2	2 (17%)	5 (46%)
	3	1 (8%)	-
**Multiparity**	Yes	-	2 (18%)
	No	12 (100%)	9 (82%)
**Gestational age at birth (weeks)**		38.2 ± 0.8	36.7 ± 4.0
**Type of delivery**	Vaginal	8 (67%)	7 (64%)
	Caesarean section	4 (33%)	4 (36%)
**Presentation**	Cephalic	11 (92%)	8 (73%)
	Breech	1 (8%)	3 (27%)
**Assisted delivery**	Yes	1 (10%)	1 (11%)
	No	9 (90%)	8 (89%)
**Birth weight (g)**		3298 ± 331	2705 ± 858
**APGAR^3^**	Normal	10 (83%)	6 (67%)
	Abnormal	2 (17%)	3 (33%)
**Developmental delay**	Yes	5 (42%)	2 (18%)
	No	7 (58%)	9 (82%)
**History of congenital torticollis**	Yes	10 (83%)	10 (91%)
	No	2 (17%)	1 (9%)
**Age when plagiocephaly was noticed (months)**		1.5 ± 1.3	1.3 ± 0.8
**Ethnicity**	Caucasian	6 (50%)	6 (67%)
	Arab/North-African	1 (8%)	2 (22%)
	Latin-American/Hispanic	1 (8%)	1 (11%)
	East-Asian mixed	2 (17%)	-
	Other/Multiple	2 (17%)	-
**Familial income**	< $80,000	1 (13%)	1 (13%)
	$80,000 – $100,000	2 (25%)	1 (13%)
	> $100,000	5 (63%)	6 (75%)
**Highest level of parental education**	High School	3 (27%)	-
	College, CEGEP	1 (9%)	3 (33%)
	University	7 (64%)	6 (67%)

1. Categorical data: n (proportion %). Percentages are calculated based on the number of available responses for each item. Missing data are due to one participant withdrawal in the control arm prior to receiving the allocated intervention and prior to most data collection, and occasional non-response in parental questionnaires. Continuous data are presented as mean ± standard deviation.

2. Birth rank: order of birth; 1 = first-born, 2 = second-born, etc.

3. APGAR score assessed at 1, 5 and 10 minutes after birth (Appearance, Pulse, Grimace, Activity, and Respiration), scored from 0 to 2 for each component, with a total score ranging from 0 to 10. Scores were grouped as either Normal (7 to 10) or Abnormal (0 to 6).

### Feasibility

Feasibility was assessed using structured stakeholder logs. Common issues, ranked by frequency, included: (**1**) logistical challenges, (**2**) data collection and measurement validity, (**3**) eligibility and recruitment, (**4**) consent procedures, and (**5**) randomization. Parental acceptability was evaluated through questionnaires.

### Logistical Challenges

Logistical challenges primarily involved coordination between orthotist and physician appointments (**Table 3**).

**Table 3:** Discrepancies between planned and actual appointment timing.

Timepoint	Median delay, days (range)	Seen on planned date, %	Seen within 1 week of planned date, %
6-week follow-up	5 days (range 0–30)	13%	65%
12-week follow-up with physician	0 day (range 0–35)	57%	57%
12-week follow-up with orthotists	6 days (range 0–28)	17%	56%
Cranial remolding orthosis delivery	1 day (range 0–42)	30%	73%

Barriers included limited orthotist availability, scheduling conflicts, and insufficient advance notice for CRO fittings, particularly for participants requiring accelerated treatment. Space constraints in clinic settings further contributed to delays and increased waiting times.

Only 30% completed both visits on the same day at 12 weeks, increasing travel and time demands.

Facilitators included early scheduling of appointments and use of dedicated clinic spaces, both of which improved protocol adherence and reduced delays.

### Eligibility Criteria and Recruitment

Initially, eligibility was based on age at treatment onset, which unintentionally excluded most control arm infants who would exceed the age limit before treatment initiation. This was amended, with ethics approval, to define eligibility based on age at initial evaluation.

Restricting enrollment to severe DP slowed recruitment. Despite these challenges, recruitment was completed within seven months.

### Data Collection and Measurement Validity

Technical and procedural challenges emerged during data collection. The Rodin4D software did not default to the 40° diagonal angles specified in the protocol for ODDI. The manufacturer confirmed the software plots the longest and shortest diagonals first and assigns remaining diagonals at a preset angle that can be manually changed to 40°. Because re-tracing all scans was beyond the scope of this pilot, ODDI values were calculated using software-generated angles (typically 29–35°).

Additional variability arose from parent misinterpretation of questionnaire items and delayed questionnaire completion when therapy extended 12 weeks, sometimes requiring later administration by phone or email, increasing time burden and recall bias. One questionnaire was lost during this stage. One follow-up visit was conducted by an orthotist unfamiliar with the study protocol, resulting in missing data at the 6-week time-point. Despite these issues, no participants were lost to follow-up.

### Consent Procedure

Most parents were enthusiastic about participating, and no family declined participation. Consent was initially planned to be obtained by dedicated support personnel; however this was not feasible, and responsibility shifted to investigators and clinicians uninvolved in direct patient care. Limited space made it difficult to allocate private rooms for both medical assessments and obtaining consent. Additionally, miscommunication posed issues; one family mistook consent as guaranteed participation, causing frustration when their child was excluded due to insufficient severity of DP. Clearer explanations were subsequently implemented.

### Randomization

Allocation concealment and assessor blinding were maintained despite limited research personnel.

One protocol deviation occurred when a participant randomized to the delayed arm received the CRO earlier than scheduled due to a scheduling error during appointment coordination.

### Parental Acceptability of Treatment

Cranial remolding orthosis was generally well tolerated. Among 23 infants, 12 required additional orthotist visits, primarily due to cranial growth requiring a new orthosis, either during the initial 12-week period or later in the treatment course. In two cases (8.7%), families needed extra visits due to improper helmet fit upon initial delivery. While these additional appointments added logistical demands, families adapted well.

All parents reported at least one side effect. The most common were:

- Sweating (n = 14),
- Helmet odor (n = 14),
- Skin irritation without wounds (n = 12),
- Sleeping issues (n = 7),
- Fit issues (n = 3),
- Baby trying to remove the helmet (n = 2),
- Pain (n = 1),
- Problems accepting the helmet (n = 1).

No wounds or skin infections were reported. Most side effects occurred when the helmet became too small, emphasizing the importance of timely follow-up. All parents stated they would repeat the treatment if needed, suggesting side effects were minor and outweighed by perceived benefits.

### Trends of Efficacy

As a feasibility study, this trial was not powered to detect statistically significant differences in treatment efficacy. Trends in cranial asymmetry were explored descriptively, with P-values and effect si es (partial η²) provided to support interpretation (**Table 4**).

**Table 4:** Changes in cranial symmetry outcomes over time (intention-to-treat analysis).

Outcome	Timepoint	Intervention (n=12)	Control (n=11)	*p* (time)	*p* (group)	*p* (group × time)	η^2^ (interaction)
**CVA (mm)**				**<0.001**	**0.200**	**0.063**	**0.138**
	Baseline	21.0 ± 5.3 (13.0–29.0)	24.2 ± 6.4 (16.0–38.0)				
	6 weeks	21.7 ± 5.9 (13.0–32.0)	27.1 ± 6.1 (19.0–39.0)				
	12 weeks	23.6 ± 5.7 (15.0–32.0)	26.1 ± 7.2 (17.0–39.0)				
	Δ Baseline → 12 weeks	2.5	1.4				
**CVAI (%)**				**0.082**	**0.194**	**0.120**	**0.108**
	Baseline	16.6 ± 4.6 (9.8–23.6)	19.6 ± 6.3 (12.2–34.9)				
	6 weeks	16.8 ± 5.0 (9.6–25.4)	21.3 ± 5.6 (14.2–33.6)				
	12 weeks	17.9 ± 4.7 (10.9–25.4)	20.4 ± 6.6 (12.4–33.9)				
	Δ Baseline → 12 weeks	1.3	0.2				
**ODDI (%)**				**<0.001**	**0.037**	**0.846**	**0.009**
	Baseline	107.6 ± 2.6 (103.5–111.1)	109.7 ± 3.3 (105.7–114.9)				
	6 weeks	105.8 ± 2.5 (102.8–109.7)	108.2 ± 3.6 (102.0–114.2)				
	12 weeks	103.9 ± 2.4 (101.3–109.7)	105.8 ± 2.4 (100.7–109.7)				
	Δ Baseline → 12 weeks	−3.5	−3.5				
**CI (%)**				**<0.001**	**0.825**	**0.294**	**0.063**
	Baseline	90.9 ± 4.5 (86.2–100.0)	92.9 ± 9.5 (77.4–109.8)				
	6 weeks	89.8 ± 4.8 (83.2–98.6)	88.1 ± 5.8 (76.1–97.2)				
	12 weeks	87.8 ± 3.7 (83.2–94.0)	87.2 ± 5.1 (76.6–93.8)				
	Δ Baseline → 12 weeks	−2.9	−4.8				

**Note:** Data are presented as mean ± standard deviation (range: min–max). η^2^ partial eta squared from mixed-model A OVA (group × time). Δ within-group change from baseline to 12 weeks (mean at 12 weeks minus mean at baseline). p-values <0.001 are reported as <0.001; other p-values are reported to three decimals.

Repeated-measures ANOVA revealed significant main effects of time for CVA, ODDI, and CI (all p < 0.001), indicating overall improvement across the study period regardless of treatment group. No significant main effect of time was observed for CVAI (p = 0.082).

A significant main effect of treatment group was observed only for ODDI (p = 0.037), suggesting an overall difference between groups across time. However, group × time interaction effects were not statistically significant for any outcome (CVA: p = 0.063; CVAI: p = 0.120; ODDI: p = 0.846; CI: p = 0.294), indicating similar trajectories of improvement in both treatment arms.

Post-hoc pairwise comparisons indicated significant improvements in CVA and ODDI from baseline to both 6 and 12 weeks (all p < 0.01). For CVA, no additional change was observed between 6 and 12 weeks. ODDI continued to improve between 6 and 12 weeks (p < 0.01). CI improved significantly between baseline and 6 weeks, with sustained improvement at 12 weeks and no further change between 6 and 12 weeks. Given the non-significant main effect of time, post-hoc comparisons were not performed for CVAI. Overall, these findings indicate that cranial symmetry improved over time in most measures, without evidence of differential rates of improvement between treatment groups.

Results from the per-protocol analysis were consistent with the intention-to-treat findings, showing similar patterns of improvement over time and no significant group × time interactions across outcomes (Appendix C, Table C1).

Clinical changes in cranial asymmetry severity, assessed by the Argenta classification, were analyzed using a Mann–hitney U test for continuous change and a χ² test with Monte Carlo-simulated p-values for categorical severity levels. The magnitude of change in Argenta score from baseline to 12 weeks did not differ significantly between groups on rank-based testing (W = 41.5, p = 0.129), indicating no statistically significant between-group difference. Descriptively, a greater number of infants in the intervention arm were classified as mild or resolved at 12 weeks compared to the control arm (four versus one).

Parent-reported outcomes included two domains. First, a head-shape perception item captured parents’ judgment of the child’s present cranial appearance. Scores improved significantly from baseline to 12 weeks in both groups (control: Wilcoxon signed-rank, V = 0, p = 0.008; intervention: V = 0, p = 0.002), indicating significant within-group improvement over time. The magnitude of change from baseline to 12 weeks did not differ significantly between groups (Mann–Whitney U, W = 59.5, p = 1.000).

Second, a treatment-satisfaction item assessed parents’ overall evaluation of the helmet-therapy experience. At 12 weeks, parent-reported satisfaction was high in both groups (control: mean ± SD, 4.27 ± 0.65; intervention: 4.42 ± 0.67). Satisfaction scores did not differ significantly between the two arms (Mann–Whitney U test, p = 0.584).

Overall, parents perceived cosmetic improvement in head shape and reported high satisfaction, independent of intervention timing.

## DISCUSSION

This pilot RCT demonstrated the feasibility and acceptability of conducting a larger-scale study evaluating CRO in infants with severe DP. All predefined feasibility objectives were met, including successful recruitment, retention, and protocol adherence. Recruitment was completed within seven months, despite restrictive age and severity inclusion criteria, supporting feasibility of enrolling this population in a future definitive trial.

Our study addresses an important gap in the existing literature, as the only prior RCT evaluating the efficacy of CRO excluded infants with the most severe deformities, prematurity, and congenital torticollis, groups that may stand to benefit most from treatment.^16^ In contrast, 83% of infants in our cohort had a history of congenital torticollis, which is comparable to findings from the prospective study by Rogers et al, in which 92% of infants with plagiocephaly demonstrated a preferential head position and 97% exhibited rotational asymmetry on physical examination.^24^ In addition, 21% in our cohort were born preterm, a group excluded from both the HEADS trial and large retrospective cohorts.^16,18^ By including infants with torticollis, very severe DP, and prematurity, our study addresses populations underrepresented in prior randomized trials and improves the generalizability of findings.

A key strength was the detailed assessment of coordination demands between orthotist and physician appointments. Prior CRO trials have focused on clinical outcomes, with limited reporting of logistical barriers. Only 30% of participants completed orthotist and physician visits on the same day at 12 weeks, highlighting an actionable target for future trials. Improved synchronization could reduce family burden and support follow-up adherence.

Cranial remolding orthosis was generally well tolerated in our cohort, and parental acceptability was high despite frequent minor side effects. All families indicated they would choose CRO again if needed, reflecting high perceived benefit and acceptance of treatment. Fewer than 10% of infants required additional adjustments due to fit issues, a lower proportion than reported in the HEADS trial.^16^ Previous studies have similarly reported mostly mild adverse effects.^25^ However, parental experience and treatment burden have been inconsistently reported.^7,16^ A further facilitator to participation was that CRO was fully funded by provincial medical insurance, eliminating financial burden for families. Our findings extend the existing literature by providing a more detailed description of parental perceptions and practical challenges associated with helmet therapy, which are relevant when assessing feasibility for a future randomized trial.

Although not powered for definitive between-group comparisons, exploratory analyses revealed significant improvement in CVA, ODDI, and CI, particularly during the first six weeks. ODDI continued to improve through 12 weeks and exhibited the strongest overall between-group differences. These findings suggest that CRO is associated with measurable improvements in cranial symmetry over time, with some metrics appearing more responsive than others. In contrast, CVAI did not change significantly in the present study, which differs from findings in larger retrospective cohorts, likely reflecting greater statistical power.^18^

No significant differences were observed between immediate and delayed treatment arms in Argenta classification or in parent-reported satisfaction with head shape. Age-related effects reported in retrospective cohorts were not observed in this pilot sample.^18^ A mid-study clinical assessment at 6 weeks, using the Argenta classification and a parental perception questionnaire, could allow meaningful comparison between groups at different intervention stages. A future study would benefit from a priori power calculation.

Protocol deviations occurred when one participant received treatment earlier than scheduled, and two participants in the intervention arm did not meet the predefined helmet wear threshold of ≥ 20 hours per day, highlighting challenges related to group allocation and treatment adherence. Similar challenges related to adherence have been reported in the RCT by van Wijk et al.^16^ Future studies may benefit from a dedicated research coordinator to support appointment scheduling, monitor adherence, and ensure protocol fidelity.

To increase the likelihood of detecting measurable changes in cranial shape within this pilot sample, inclusion was restricted to severe DP. Future trials should include moderate cases to improve recruitment efficiency and generalizability, in line with current clinical practice.^15^

Several measurement-related challenges were identified. In one case, the shortest diagonal corresponded to the biparietal width rather than an oblique diagonal, inflating both CVA and CVAI values. Although CVAI, as originally described, is defined using oblique diagonals measured at a fixed angle from the anteroposterior axis, this specification is not consistently enforced across software platforms or explicitly reported in the literature.^26^ Recent work has highlighted substantial variability in how CVAI is calculated, including differences in diagonal orientation and denominator selection, underscoring the need for clearer methodological standards.^27^ Moreover, measurement consistency, particularly related to ODDI angle settings in Rodin4D software, posed challenges in this study. Future trials should standardize diagonal angles and software settings prior to data collection to improve measurement precision. These findings highlight the importance of careful outcome selection and standardized measurement procedures in future trials. In this pilot study, CVA, ODDI, and CI demonstrated consistent sensitivity to change over time, whereas CVAI appeared more susceptible to measurement variability related to diagonal orientation and software implementation. As 3D volumetric techniques become more accessible, they may offer a valuable global index of cranial symmetry.^3,28^

## CONCLUSION

This pilot RCT demonstrated the feasibility and acceptability of a larger study on CRO for infants with severe DP. Recruitment, retention, and adherence were adequate, and study procedures were successfully implemented, supported by high parental acceptance.

Although not powered to assess efficacy, exploratory analyses showed significant improvement in CVA, ODDI, and CI over time, particularly in the early weeks of treatment. No statistically significant differences were observed between early and delayed treatment arms by 12 weeks; however trends in ODDI suggested a potential treatment effect.

Future studies should address identified logistical and measurement challenges and broaden eligibility criteria to moderate cases. A dedicated research coordinator may improve scheduling efficiency, reduce participant burden, and improve data quality.

Overall, these findings support progression to a larger RCT and provide actionable guidance for future trial design evaluating the effectiveness of CRO for infants with severe DP.

## Figures and Tables

**Figure:**
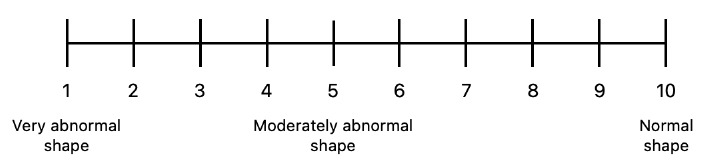


**Figure:**
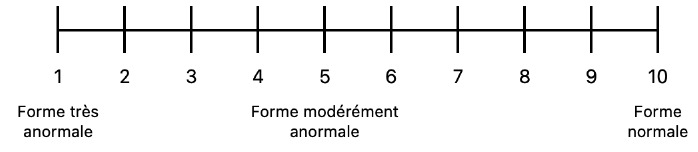


**Figure:**
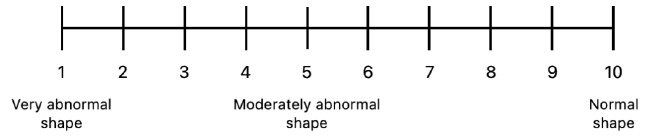


**Figure:**
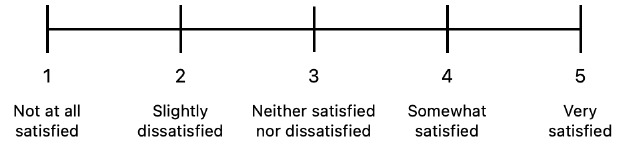


**Figure:**
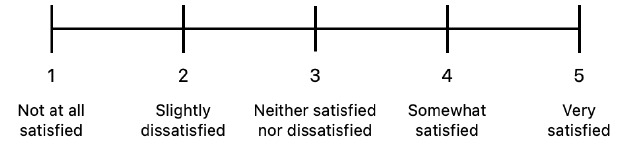


**Figure:**
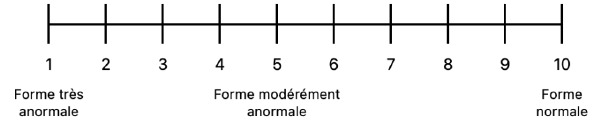


**Figure:**
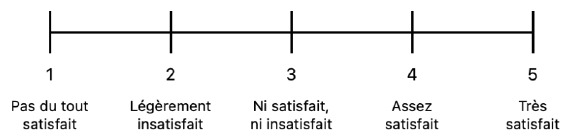


**Figure:**
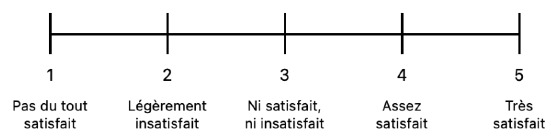


**Table B1:** Classification of head deformation.

Category	CVA (mm)	CVAI (%)	ODDI (%)	CI (%)	Argenta classification
**Normal head shape**	<3	<3	<104	<90	0
**Mild deformation**	3-10	3-7	104-107	90-94	I
**Moderate deformation**	10-12	7-12	108-111	95-99	II
**Severe deformation**	>12	>12	≥112	≥100	III-V

**Table C1:** Changes in cranial symmetry outcomes over time (per-protocol analysis).

Outcome	Timepoint	Intervention (n=11)	Control (n=10)	*p* (time)	*p* (group)	*p* (group × time)	η^2^ (interaction)
CVA (mm)				**<0.001**	**0.095**	**0.090**	**0.132**
	Baseline	20.6 ± 5.5 (13.0–29.0)	24.7 ± 6.5 (16.0–38.0)				
	6 weeks	21.8 ± 6.3 (13.0–32.0)	27.1 ± 6.1 (19.0–39.0)				
	12 weeks	22.6 ± 5.3 (15.0–32.0)	26.9 ± 7.2 (17.0–39.0)				
	Δ Baseline → 12 weeks	1.8	1.6				
CVAI (%)				**0.169**	**0.107**	**0.215**	**0.086**
	Baseline	16.3 ± 4.7 (9.8–23.6)	20.0 ± 6.5 (12.2–34.9)				
	6 weeks	16.9 ± 5.3 (9.6–25.4)	21.3 ± 5.6 (14.2–33.6)				
	12 weeks	17.2 ± 4.4 (10.9–25.4)	21.0 ± 6.7 (12.4–33.9)				
	Δ Baseline → 12 weeks	0.8	0.4				
ODDI (%)				**<0.001**	**0.078**	**0.889**	**0.007**
	Baseline	108.1 ± 2.2 (104.2–111.1)	110.0 ± 3.3 (105.7–114.9)				
	6 weeks	106.1 ± 2.5 (102.8–109.7)	108.2 ± 3.6 (102.0–114.2)				
	12 weeks	104.2 ± 2.5 (101.3–109.7)	105.7 ± 2.5 (100.7–109.7)				
	Δ Baseline → 12 weeks	−3.7	−3.9				
CI (%)				**<0.001**	**0.512**	**0.635**	**0.026**
	Baseline	92.7 ± 6.0 (86.2–104.7)	91.7 ± 9.2 (77.4–109.8)				
	6 weeks	90.1 ± 5.1 (83.2–98.6)	88.1 ± 5.8 (76.1–97.2)				
	12 weeks	88.7 ± 4.0 (83.2–94.0)	86.5 ± 4.9 (76.6–93.8)				
	Δ Baseline → 12 weeks	−3.9	−4.1				

**Note:** Data are presented as mean ± standard deviation (range: min–max). η^2^ partial eta squared from mixed-model ANOVA (group × time). Δ within-group change from baseline to 12 weeks (mean at 12 weeks minus mean at baseline). p-values <0.001 are reported as <0.001; other p-values are reported to three decimals.

## APPENDIX

### Appendix A

**PARENTAL QUESTIONNAIRES** (Pre- and post-treatment questionnaires in English)

**Study code** : ………………

(To be completed by research team)

**Date** : ………………

**PRE-TREATMENT QUESTIONNAIRE**

1. How old is your child (in months)? ………………
2. What was the gestational age of your child at the time of birth (in weeks)? ………………
3. About the delivery:
  - Type of delivery (vaginal or cesarean): ………………
  - Baby’s presentation (cephalic or breech presentation): ………………
  - Complications during delivery: ………………
4. APGAR score at birth: ………………
5. Birth weight: ………………
6. Was this your first delivery? If not, how many deliveries have you had before? ………………
7. Does your child have any medical conditions? If so, what are they? ………………
8. Did your child have a torticollis during the first months of his life? ………………
9. How would you rate **the shape of your child’ head**, on a scale of 1 to 10? *(Circle your answer)*
10. How old was your child when you or your doctor noticed the asymmetry of the head? ………………
11. Has your child tried any other interventions to treat the asymmetry of the head (E.g. physical therapy, osteopathy, etc.) ………………
12. Does your child tend to turn his/her head to one side more than the other when sleeping?
  - To the right side
  - To the left side
  - Flat on the back
  - No preferred side noticed
13. Additional comments: ………………

**PARENTAL QUESTIONNAIRES** (Pre- and post-treatment questionnaires in French)

**Code de recherche** : ………………

(À compléter par l'équipe de recherche)

**Date** : ………………

**QUESTIONNAIRE PRÉ-TRAITEMENT**

1. Quel âge a votre enfant (en mois)? ………………
2. Votre enfant est né à combien de semaines de gestation? ………………
3. Informations sur l’accouchement
  - Type d’accouchement (par voie vaginale ou par césarienne)
  - Position du bébé (présentation céphalique ou par siège):
  - Complications à l’accouchement?
4. Score d’APGAR à la naissance : ………………
5. Poids à la naissance : ………………
6. Est-ce qu’il s’agissait de votre premier accouchement? Sinon, combien d’accouchements ave-vous eu auparavant? ………………
7. Votre enfant a-t-il des problèmes de santé? Si oui, lesquels? ………………
8. Votre enfant a-t-il eu un torticolis durant ses premiers mois de vie? ………………
9. Comment qualifiez-vous **la forme de tête de votre enfant**, sur une échelle de 1 à 10? *(Encerclez votre réponse) :*
10. Quel âge avait votre enfant lorsque vous ou votre médecin a remarqué l’asymétrie de la tête? ………………
11. Votre enfant a-t-il essayé d’autres interventions pour traiter cette asymétrie de la tête? (Exemple : physiothérapie, ostéopathie, etc.) ………………
12. Votre enfant a-t-il tendance à tourner sa tête d’un côté plus que de l’autre lorsqu’il dort?
  - Vers le côté droit
  - Vers le côté gauche
  - Sur le dos
  - Aucun côté préférentiel
13. Commentaires supplémentaires : ………………

**POST-TREATMENT QUESTIONNAIRE** (To be completed by research team)

**Study code** : ………………

**Date**: ………………

1. Your child has worn the cranial orthosis between the following dates:
  ______ /______ /______ to ______ /______ /______.
2. On average, how many hours per day did your child wear the cranial orthosis? ………… If it was less than 20 hours, why? …………
3. Did you notice any side effects while your child was wearing the cranial orthosis? Check all that apply:
  - No side effects
  - More frequent crying
  - Difficulty sleeping
  - Redness and irritation of the skin
  - Sores/wounds
  - Skin infection
  - Excessive sweating
  - Unpleasant odor of the cranial orthosis
  - If you checked one or more side effects, please describe the issue:
  - Other side effects:
4. Have you had any problems with the fit of the helmet that required an additional visit with the orthotist?
5. Did your child receive any other interventions during the treatment period (E.g. physiotherapy, osteopathy, etc.) ………………
6. How would you rate **your child’s current head shape**, on a scale of 1 to 10? *(Circle your answer)*
7. How satisfied are you with **your child’s head shape** following treatment? *(Circle your answer)*
8. How satisfied are you with **the treatment by cranial orthosis (helmet)**? *(Circle your answer)*
9. Do you agree with the following statement:
  ***I would agree to repeat cranial orthosis therapy with another child of mine if needed** (yes or no)*? ………
10. Do you have additional comments on the treatment or on the procedure employed in this research project? ………………

**The following questions are for statistical research purposes. Answers to these questions are optional.**

11. What is your child’s ethnicity?
12. In what annual salary range (gross) is your family?
  - Less than $10,000
  - From $10,000 to less than $20,000
  - From $20,000 to less than $30,000
  - From $30,000 to less than $40,000
  - From $40,000 to less than $50,000
  - From $50,000 to less than $60,000
  - From $60,000 to less than $70,000
  - From $70,000 to less than $80,000
  - From $80,000 to less than $90,000
  - From $90,000 to less than $100,000
  - More than $100,000
13. What is the highest level of education completed by you or your spouse?
  - Elementary school
  - High school
  - College/CEGEP
  - University
  - Other

**QUESTIONNAIRE POST-TRAITEMENT** (À compléter par l’équipe de recherche)

**Code de recherche** : ………………

**Date**: ………………

1. Votre enfant a porté l’orthèse crânienne entre les dates suivantes : ______ / ______ / ______ à ______ / ______ / ______.
2. En moyenne, combine d’heures par jour votre enfant a-t-il porté l’orthèse? ………………
  Si moins de 20 heures, pour quelle raison? ………………
3. Avez-vous noté des effets secondaires lors du port de l’orthèse crânienne? Coche tout ce qui s’applique
  - Aucun effet secondaire
  - Pleurs plus fréquents
  - Difficultés à dormir
  - Rougeur et irritation de la peau
  - Plaies
  - Infection de la peau
  - Transpiration excessive
  - Odeur désagréable de l’orthèse crânienne
  - Si vous avez coché un effet secondaire ou plus, veuillez décrire la problématique: …………
  - Autres effets secondaires:
4. Avez-vous eu des problèmes avec l’ajustement du casque ayant nécessité une visite supplémentaire avec l’orthésiste? …………
5. Votre enfant a-t-il bénéficié d’autres interventions durant la période de traitement? (Exemple : physiothérapie, ostéopathie, etc.)
6. Comment qualifiez-vous **la forme de tête de votre enfant actuellement**, sur une échelle de 1 à 10 ? *(Encerclez votre réponse)*
7. Quelle est votre satisfaction avec **la forme de tête de votre enfant suite au traitement**? *(Encerclez votre réponse)*
8. Quelle est votre satisfaction avec **le traitement par orthèse crânienne (casque)**? *(Encerclez votre réponse)*
9. Êtes-vous d’accord avec l’énoncé suivant;
  **J’accepterais de refaire un traitement par orthèse crânienne avec un autre de mes enfants au besoin** *(oui ou non)?* ………………
10. Avez-vous des commentaires sur le traitement ou sur la procédure de ce projet de recherche? ………………
  **Les questions suivantes sont à des fins de statistiques pour la recherche. Les réponses sont optionnelles.**
11. Quelle est l’origine ethnique de votre enfant? ………………
12. Dans quel échelon salarial annuel (brut) votre famille se situe-t-elle?
  - Moins de 10 000$
  - De 10 000$ à moins de 20 000$
  - De 20 000$ à moins de 30 000$
  - De 30 000$ à moins de 40 000$
  - De 40 000$ à moins de 50 000$
  - De 50 000$ à moins de 60 000$
  - De 60 000$ à moins de 70 000$
  - De 70 000$ à moins de 80 000$
  - De 80 000$ à moins de 90 000$
  - De 90 000$ à moins de 100 000$
  - Plus de 100 000$
13. Quel est le plus haut niveau de scolarité complété par vous ou votre conjoint(e)?
  - Études primaires
  - Études secondaires
  - Études collégiales/CÉGEP
  - Études universitaires
  - Autres: ____________

### Appendix B

**CRANIAL MEASUREMENTS**

The **cranial vault asymmetry (CVA)** is the difference between the longest and the shortest cranial diagonals, in millimeters.^29^ However, authors are inconsistent regarding cut-off values for classification.^30^ The most rigorous classification has been described by Hutchison, et al., in studies published in 2005 and 2009, as they correlated each level of severity with response to treatment (see Table B1 for severity classification).^31,32^

Eq. (A.1): CVA = longest diagonal – shortest diagonal

The **cranial vault asymmetry index (CVAI)** is a measure of CVA considering the overall size of the head, to obtain a percentage value.^27,28^

Eq. (A.2): CVAI = (longest diagonal – shortest diagonal) / shortest diagonal x 100

The **oblique diameter difference index (ODDI)**, also called oblique cranial length ratio (OCRL), provides the degree of the plagiocephalic component of the head deformation.^33^ It is a ratio between the longest cranial diagonal and the shortest cranial diagonal multiplied by 100, with both diagonals measured 40 degrees from the anterior-posterior line. A head shape that is perfectly symmetrical will have a 100% ODDI; a value of more than 100% represents asymmetrical head deformation. There is a discrepancy in the cut-off values used for classification, but we used the ones used in the RCT conducted by van Wijk et al. (see Table B1).^16^

Eq. (A.3): ODDI = (longest diagonal / shortest diagonal) x 100

The **cranioproportional index (CI)** is the ratio between the width (medio-lateral line) and the length (anterior-posterior line) of the skull and determines the brachycephalic component of the deformation. A score of 80% on the cranioproportional index represents an average head shape in Western countries. A higher value represents a larger head width compared with length.^33^

Eq. (A.4): CI = head width / head length x 100

The Argenta classification system was used as described by Argenta.^21^ The Argenta classification was chosen as a pragmatic clinical tool with documented moderate inter-rater reliability and good intra-rater reliability when used by clinicians routinely involved in the assessment of deformational plagiocephaly.^34^

### Appendix C

**CHANGES IN CRANIAL SYMMETRY OUTCOMES OVER TIME, IN PER-PROTOCOL ANALYSIS**
